# What complex factors influence sleep quality in college students? PLS-SEM vs. fsQCA

**DOI:** 10.3389/fpsyg.2023.1185896

**Published:** 2023-08-25

**Authors:** Ying Wang, Xinyi Dai, Jie Zhu, Zeling Xu, Jiayao Lou, Keda Chen

**Affiliations:** Shulan International Medical College, Zhejiang Shuren University, Hangzhou, China

**Keywords:** sleep quality, stress, self-control, qualitative comparative analysis, structural equation models

## Abstract

**Introduction:**

Sleep quality has a significant impact on the health-related quality of life, particularly among college students. This study proposes a framework for identifying factors that influence college students’ sleep quality, including stress, self-control, bedtime habits, and neighborhood environment.

**Methods:**

The study employed a cross-sectional analytical approach on a convenience sample of 255 medical students from a private university in China during the 2021/2022 academic year, of which 80.39% (205) were women. Two complementary methodologies, partial least squares-structural equation modeling (PLS-SEM), and fuzzy sets qualitative comparative analysis (fsQCA), were utilized in the study.

**Results:**

The results of the PLS-SEM analysis suggest that Stress and Self-control act as mediating variables in the model, with Bedtime habits and Neighborhood environment influencing sleep quality through these variables. Additionally, the fsQCA analysis reveals that Bedtime habits and Neighborhood environment can combine with Stress and Self-control, respectively, to influence sleep quality.

**Discussion:**

These findings provide insight into how multiple factors, such as Stress, Self-control, Bedtime habits, and Neighborhood environment, can impact college students’ sleep quality, and can be used to develop intervention programs aimed at improving it. Moreover, the use of both methodologies enables the expansion of new methodological approaches that can be applied to different contexts.

## 1. Introduction

Sleep occupies one-third of a person’s life (Bixler, 2009), but with the accelerating pace of work and life, the incidence of sleep disorders is increasing every year. Approximately 45.4% of adults in China have experienced or are currently experiencing sleep disorders (Zhang et al., 2018). One of the significant challenges that higher education institutions face today is the issue of sleep sub-health among college students. Recent evidence indicates that medical students are facing a lack of sufficient sleep, with an average duration of 6.3 h per night. Additionally, 55% of the students exhibited poor sleep quality based on the Pittsburgh Sleep Quality Index (PSQI), while 31% experienced excessive daytime sleepiness as assessed by the Epworth Sleepiness Scale (ESS) (Jahrami et al., 2020). Poor sleep quality has a considerable impact on the regular study and daily life of college students and can even lead to major events. Sleep disorders among college students are mainly characterized by a constantly disrupted biological clock, resulting in delayed bedtimes and irregular sleep patterns. Chronotype serves as a robust predictor of sleep quality, indicating that evening-type students could enhance their sleep quality by adjusting their schedule to align with earlier hours (Sun et al., 2019). The group-level impact of these issues is significant. Lack of sleep can lead to physical and mental sub-health, as well as academic performance decline (Owens et al., 2017). Therefore, actively researching the factors that influence students’ sleep quality is of practical significance in improving their overall sleep quality.

The factors that impact sleep quality are diverse and varied (Wang and Bíró, 2021), including sociodemographic factors, lifestyle habits, health status, psychological conditions, and environmental factors (Karakan, 1983). A study conducted on the factors influencing sleep quality among students identified several related factors contributing to sleep disorders, such as irregular sleep patterns, self-perceived health status, academic and job-related stress, and dormitory environment (Zheng et al., 2016). Age, perceived stress levels, pain tolerance, smartphone addiction, and moderate physical activity are among the multiple variables that can affect sleep quality (Kumar et al., 2019; Cavic et al., 2021; Memon et al., 2021). Furthermore, chronic physiological conditions such as diabetes, heart disease, menopause, and dietary habits also have a certain impact on sleep quality (Tasdemir and Oguzhan, 2016). These findings highlight the complexity and multifaceted nature of sleep quality, involving multiple factors that interact with each other. Therefore, it is essential to explore the various complex factors and examine the mediating role of each factor, as well as the influence of their complex interactions on sleep quality. This approach provides a valuable reference for understanding the complex factors that affect sleep quality and developing effective strategies to improve it. Research has indicated a noteworthy negative correlation between sleep quality and stress levels (Alotaibi et al., 2020), where reducing stress can lead to improved sleep quality, while heightened stress can severely degrade it (Van Laethem et al., 2017). Additionally, there is a positive correlation between sleep quality and self-control (Guarana et al., 2021). Unhealthy bedtime habits, such as the use of blue light-emitting devices before sleep, have been found to be significantly associated with poor sleep quality (Jniene et al., 2019). Furthermore, the duration of mobile phone use before bedtime has a negative correlation with the sleep quality of adults (Exelmans and Van den Bulck, 2016). The quality of sleep is significantly impacted by the surrounding environment. For example, a cold indoor environment can have a negative effect on sleep quality, whereas a warm and comfortable sleeping environment can improve it. Therefore, improving environmental factors can lead to enhanced sleep quality (Billings et al., 2020). However, these studies often focus on single factors that affect sleep quality, while in reality, multiple factors influence sleep quality in complex and multifaceted ways, and there may be significant interactions between these factors. For college students, psychological pressure and self-control play a dominant role in their daily learning and life (Peng et al., 2022). Moreover, the dormitory environment is distinct from that of the wider community, as it is more homogeneous and easily influenced by the sleep habits of individuals and others. Research has found a strong correlation between perceived stress, mobile phone addiction, and low self-control (Zhang et al., 2022). As sleep disorders among college students have been linked to stress-induced depression, there is a need to investigate how these factors interact and impact sleep quality (Peltz and Rogge, 2016). Hence, this study aims to explore the complex interplay between these factors and their influence on sleep quality.

This study aimed to evaluate the factors (stress, self-control, bedtime habits, neighborhood environment) that affect college students’ sleep quality using two different approaches: partial least squares structural equation modeling (PLS-SEM) and qualitative comparative analysis (QCA).

## 2. Theoretical framework and hypothesis development

### 2.1. PLS-SEM model

**Figure 1:**
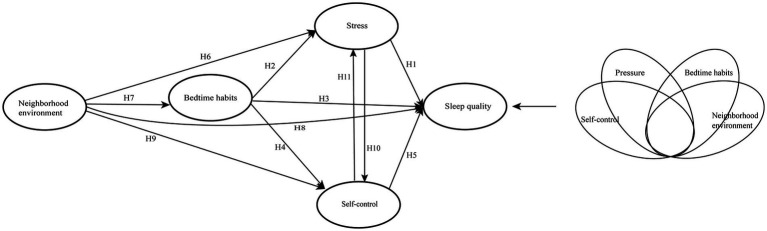
Conceptual model. On the left is the conceptual model of PLS-SEM, and on the right is the conceptual model of fsQCA.

Prior research has established that stress (Brand et al., 2010), self-control (Brando-Garrido et al., 2022), bedtime habits (Dinis and Bragança, 2018), and neighborhood environment (Billings et al., 2020) are significant factors that impact sleep quality. The proposed model posits that these factors have a direct effect on sleep quality, with stress, self-control, and sleep habits playing a mediating role in the relationship between these influencing factors and sleep quality (Voinescu and Szentagotai-Tatar, 2015). Notably, stress and self-control may have a dual mediating effect (Dewald et al., 2014; Figure 1 and Table 1).

**Table 1:** Hypotheses based on theory.

Hypothesis	The hypothetical path of the theoretical framework
H1	Stress had a direct effect on sleep quality.
H2	Bedtime habits had a direct effect on stress.
H3	Bedtime habits had a direct impact on sleep quality.
H4	Bedtime habits had a direct effect on self-control.
H5	Self-control had a direct effect on sleep quality.
H6	Neighborhood environment had a direct effect on stress.
H8	Neighborhood environment had a direct effect on sleep quality.
H9	Neighborhood environment had a direct effect on self-control.

#### 2.1.1. Stress

Stress is a significant societal issue in modern times. Chronic exposure to psychological stress has been associated with dysregulation of multiple physiological systems, and there is a link between mental stress and diseases such as coronary artery disease (Wei et al., 2014) and metabolic syndrome (Bergmann et al., 2014). Stress can also lead to sleep disorders (Deguchi et al., 2017), with perceived stress being a key antecedent of poor sleep quality. When attempting to fall asleep, individuals often experience worries about the future and reflect on the events of their daily lives. Bedtime anxiety is commonly comorbid with stress (Brand et al., 2010). In the workplace, potential stressors for employees include work demands, job insecurity, intra-group conflict, work-related stress, effort-reward imbalance, employment level, and shift work (Deguchi et al., 2017). For students, stress levels often peak during exam periods. Studies have shown that students experience more fragmented sleep during times of increased stress, indicating that they experience more restlessness during periods characterized by higher levels of stress (Dewald et al., 2014). Exam stress can also result in reduced cardiopulmonary function during sleep (Sakakibara et al., 2008), further impacting the quality of sleep for students. Additionally, sleep deprivation can act as a source of stress (Akerstedt, 2006) and lead to more severe psychological and physical issues, including behavioral problems, irritability, dysphoric moods, impaired cognitive and learning performance, and even harmful neurobiological changes (Curcio et al., 2006). On this basis, the hypotheses 1 are proposed (Table 1).

#### 2.1.2. Self-control

Self-control refers to the ability to regulate oneself based on goals, priorities, and environmental demands (Tangney et al., 2004), and is considered a key psychological mechanism. Self-efficacy and beliefs play a crucial role in self-control, as they influence people’s feelings, thoughts, motivation, and behavior. Good self-control has been associated with better academic performance, greater adaptability, stronger interpersonal skills, and improved emotional well-being, while poor self-control has been linked to negative outcomes in areas such as academic performance, social life, personal adaptation, and emotions (Tangney et al., 2004). In terms of sleep, good self-control is considered a protective factor against bedtime procrastination, depression, and anxiety (Geng et al., 2021), as people with better self-control tend to experience less bedtime procrastination and report better sleep quality (Brando-Garrido et al., 2022). Hence, the hypotheses 5 are proposed (Table 1).

#### 2.1.3. Bedtime habit

Sleep habits refer to a series of sleep-related behaviors, including sleep–wake behaviors, sleep schedule, sleep environment, and electronic media use, among others (Jones et al., 2020). In contemporary college students, irregular bedtime and poor sleep quality are common issues (Kabrita et al., 2014), with insomnia and staying up late being prevalent (Lund et al., 2010). Many students also have the habit of using mobile phones before sleep, which has been shown to be negatively associated with sleep quality in adults (Exelmans and Van den Bulck, 2016). Mobile phone dependence and exposure to blue light have been linked to sleep and obesity problems (Thomée, 2018), and reduced sleep has been found to lead to a decline in academic performance (Yeluri et al., 2021). Studies have shown that restricting college students’ electronic media use before bedtime could improve sleep quality to some extent (Wang et al., 2019). Sleep habit is an aspect of sleep hygiene belief, and the better the sleep hygiene belief, the higher the sleep quality (Dinis and Bragança, 2018). Therefore, we put forward the hypotheses 3 (Table 1).

#### 2.1.4. Neighborhood environment

The neighborhood environment can be divided into two parts: social environment and physical environment (Johnson et al., 2017). A positive correlation has been found between the neighborhood environment and sleep quality. Research has shown that a negative assessment of the neighborhood environment is associated with poor sleep quality (Brouillette et al., 2011), and exposure to excessive light or community noise at night is associated with lower sleep efficiency (Billings et al., 2020). A lower sense of neighborhood safety can lead to poor sleep quality and even insomnia (Hill et al., 2016). Traffic noise has also been associated with poor sleep quality or reduced sleep time (DeSantis et al., 2016). For college students, the physical environment of the neighborhood refers to the campus dormitory environment, which has a relatively simple effect on sleep quality. However, the influence of the social environment in the neighborhood, such as the influence of roommates, is relatively complex and cannot be ignored. The social environment of the neighborhood, including noise (such as electronic media and speech) and lighting in the dormitory, can affect the sleep quality of college students (Peltz and Rogge, 2016). The hypothesis 8 is therefore formulated (Table 1).

#### 2.1.5. Mediating effect

High levels of psychological stress can negatively affect the sleep quality of college students (Alsaggaf et al., 2016). The interaction between sleep quality and trait self-control suggests that lower self-control can lead to lower sleep quality (van Eerde and Venus, 2018). Bedtime habits, including the use of electronic devices before bedtime, can also affect sleep quality to a certain extent (Mireku et al., 2019). While the use of electronic devices may provide temporary relief from psychological pressure, it can also interfere with healthy sleep patterns (Ozcan and Acimis, 2021). The neighborhood environment, which includes the personal sleeping environment and the sleep habits of other members in the same dormitory, can also have an impact on sleep quality. Personal sleep cognition and sleep aids are important long-term sleep habits that can affect sleep quality. Environmental disturbances and poor sleep hygiene habits have been found to be associated with poor sleep quality in individuals with low self-control (Lin and Chung, 2022), which in turn can lead to increased psychological stress (Aizawa and Omori, 2021). Additionally, the sleep patterns and habits of other members in the environment can also affect an individual’s sleep quality through the process of operational conditioning reflex. When the behavioral events of other members in the environment are positive and valued, individuals are more likely to engage in similar behaviors, which can affect their self-control and ultimately their sleep quality (Jiang et al., 2020). Several studies have suggested that individuals may engage in behavior changes, such as increased social media use, as a coping mechanism to deal with stress (Jeong et al., 2019). However, other studies have shown that changing one’s cognitive and behavioral responses to stress and regulating negative emotions can effectively mitigate the negative impact of stress (Hamama et al., 2000). People’s emotional stress plays a mediating role in the residential environmental health and the sleep quality (Qu et al., 2023). Self-control also mediates between traitic anxiety and bedtime procrastination (Zhang et al., 2023). Therefore, it is possible that both stress and self-control may have mediating effects on sleep quality. By examining the conditions under which psychological stress and self-control influence sleep quality, we can identify ways to improve sleep quality in individuals. Hence, in line with the extensive literature, we propose hypothesize 2, hypothesize 4, hypothesize 6 and hypothesize 9 (Table 1).

### 2.2. fsQCA model

To explore the complex causal relationships that affect sleep quality and predict the combination of factors that influence sleep quality, we utilized the fsQCA configuration model. Figure 1 provides a theoretical framework that illustrates the relationships in our study, based on both theoretical and empirical background.

## 3. Materials and methods

### 3.1. Participants and data collection

A cross-sectional analytical study was conducted on a convenience sample of 255 students (Age 19–21) enrolled in the medical degree program at a private university in China, of whom 205 (80.39%) were female. All procedures in this study adhered to the World Medical Association (WMA) Declaration of Helsinki (2013) ethical guidelines, with a focus on ensuring the anonymity, confidentiality, and non-discrimination of participants. The Research Ethics Committee of Zhejiang Shuren University approved this study (202201014). All participants received comprehensive information about the study’s objectives and procedures and were informed about the confidentiality of their participation. Inclusion criteria for participation in the study were the absence of significant physical illnesses and active enrollment in regular studies. Participants provided informed consent before inclusion in the study, and the confidentiality of the information they provided was respected. Data collection was conducted online during the 2021/2022 academic year, and the survey took approximately 5 min to complete.

### 3.2. Research instruments

The questionnaire used in this study included measures of antecedent and outcome variables. The antecedent variables consisted of neighborhood environment, bedtime habits, stress, and self-control, while sleep quality was considered to be a construct consisting of four antecedent variables and two dual mediators. The questionnaire (Table 2) was developed based on existing literature, and the content of the scale’s questions was translated from English to Chinese by three translators (Brislin, 1980), with minor wording modifications to suit a college population while attempting to remain faithful to the original wording.

**Table 2:** Construct items measurement model.

Constructs/Items	Outer loadings
Stress (CR = 0.806, CA = 0.705, AVE = 0.455)	
My stress in family life comes from disagreements with my parents (S1)	0.590
My stress at school comes from some difficulties in science (S2)	0.672
Among my peers, my pressure comes from not fitting in (S3)	0.672
Worrying about the future causes me stress (S4)	0.716
My stress in the conflict between study and leisure comes from not having enough leisure time (S5)	0.714
Self-control (CR = 0.874, CA = 0.711, AVE = 0.775)	
I sometimes fail to complete tasks on time (SC1)	0.875
Some people would say I’m impulsive (SC2)	0.887
Neighborhood environment (CR = 0.912, CA = 0.807, AVE = 0.837)	
My neighborhood (dormitory or residence) is friendly (ND1)	0.904
My neighborhood (dormitory or residence) around the security is good (ND2)	0.926
Bedtime habits (CR = 0.768, CA = 0.613, AVE = 0.456)	
I watch short videos before I go to bed (BH1)	0.668
I listen to music before going to bed (BH2)	0.550
I chat on my cell phone before I go to bed (BH3)	0.719
I eat before I go to bed (BH4)	0.747

CR, composite reliability; CA, Cronbach’s alpha; AVE, average variance extracted; S, stress; SC, self-control; ND, neighborhood environment; BH, bedtime habits.

Stress (five items) was assessed utilizing the Adolescent Stress Questionnaire (ASQ) (Byrne et al., 2007), initially developed to capture data on the types of stressors experienced by adolescents. The original scale showed a positive correlation with measures of anxiety and depression and a negative correlation with measures of self-esteem, indicating that they are valid indicators of adolescent stress.

Self-control (two items) was measured using the method proposed by Tangney et al. (2004). This scale represents a novel approach for assessing individual differences in self-control. The original study on the scale demonstrated that higher self-control scores were associated with higher grade point averages, improved adaptability, reduced tendencies for overeating and alcoholism, enhanced interpersonal skills, and better emotional responses.

Neighborhood environment (two items) was measured by a scale in the study conducted by Nam et al. (2018), which was also utilized to evaluate the research participants’ neighborhood environment in the original study. The original study utilized a specific scale to assess the variables, and the findings indicated that individuals with poor sleep quality were more likely to report a lower quality of the neighborhood social environment, a reduced overall neighborhood environment, heightened dysfunctional beliefs concerning sleep, and inferior sleep hygiene compared to those with good sleep quality.

Bedtime habits (four items) were measured using the Sleep Hygiene Behavior Scale proposed by Newland et al. (2019) and the Lifestyle Habits Scale proposed by Monma et al. (2018). The Sleep Hygiene Behavior Scale measured the frequency of certain behaviors performed before bed. On the other hand, studies using the Lifestyle Habits Scale have also suggested that factors such as pre-sleep smartphone usage are independently linked to sleep disorders.

All items were evaluated using a 5-point Likert scale, ranging from 1 (strongly disagree) to 5 (strongly agree), and the scores of items in reverse scoring were reversed. The study complied with the basic principles of the Helsinki Declaration, and all participants gave informed consent prior to inclusion in the study. Sleep quality was assessed using the Pittsburgh Sleep Quality Index (PSQI) (see Supplementary Material for details) (Buysse et al., 1989). The PSQI consists of 18 questions about sleep in the past month, measuring seven factors including “Sleep quality,” “Sleep latency,” “Sleep duration,” “Habitual sleep efficiency,” “Sleep disturbance,” “Use of sleep medication,” and “Daytime dysfunction.” An overall sleep quality score was calculated by summing the scores across the 7 items. Each item was scored from 0 to 3, and the total score ranged from 0 to 21 points. Higher scores indicated poorer sleep quality, with a critical value of 5.5, and scores above 5.5 indicating the presence of a “sleep disorder.” This evaluation method is used for population screening of insomnia, with a sensitivity of 85.7% and a specificity of 86.6% (Doi et al., 2000).

### 3.3. Statistical analyses

#### 3.3.1. PLS-SEM analysis

We employed partial least squares-structural equation modeling (PLS-SEM) with SmartPLS3.3.2 to investigate the unique effects of each variable on the outcome of interest. To establish the characteristics of the model, we assessed internal consistency reliability, convergent validity, discriminant validity, and composite reliability. Cronbach’s Alpha coefficient was higher than 0.6, meeting the standard recommended by Cohen (2013), It would indicate useful reliability. The average variance extracted (AVE) exceeds 0.4, indicating that it meets the acceptable threshold and the effectiveness of convergence (Fornell and Larcker, 1981). To establish discriminant validity, the average variance extracted (AVE) of each construct should be compared with the squared correlations between the construct and other constructs in the model. Discriminant validity is achieved when a latent variable explains more variance in its associated indicator variables than it shares with other constructs within the same model (Henseler et al., 2015).

Based on the hypothesized model, we assessed the direct predictive effects of stress, self-control, bedtime habits, and neighborhood environment, as well as the mediating effects of stress and self-control, on sleep quality. To test the significance of the path coefficients, we employed Bootstrapping with 5,000 sub-samples (Henseler et al., 2009; Hair et al., 2011).

#### 3.3.2. fsQCA analysis

We utilized fsQCA2.5 to conduct a fuzzy set qualitative comparative analysis (fsQCA) aimed at identifying the combination of conditions that impact sleep quality. Qualitative Comparative Analysis (QCA) is frequently employed to explore causality in situations involving complex outcomes determined by multiple factors. The fundamental principle of QCA is that diverse causal conditions or pathways can result in a singular outcome (principle of equivalence) (Hill et al., 2019). In contrast to the probabilistic approach, QCA emphasizes the identification of commonalities among cases and their association with a specific outcome. QCA combines elements of quantitative and qualitative methods and is particularly well-suited for evaluating small sample sizes (Kane et al., 2014). Hence, QCA is an appropriate approach for translational research evaluation, often employing a hybrid methodology and analyzing limited sample sizes (Green and Estabrooks, 2011). In fsQCA analysis, variables are transformed into sets to analyze the causal set combinations of the subsets that comprise the result set. To achieve this, we converted the raw data into fuzzy sets by setting the original values from the Likert scale and calibrated it following Ragin’s suggestion (Ragin, 2009), using three substantial thresholds: fully belonging (1), not belonging at all (0), and crossover point (0.5). The intersection of these thresholds represented the maximum fuzzy point. Next, we conducted a necessity analysis, which generally assumed that a condition or combination of conditions is “necessary” or “almost always necessary” when the consistency score was higher than 0.9 (Ragin et al., 2008). Finally, we performed truth table operations to generate three possible solutions for sufficient analysis: complex, parsimonious, and intermediate [with the latter being recommended by Ragin, 2009]. We evaluated raw coverage, which indicates how many cases or observations can be explained by each path (condition or combination of conditions), unique coverage, which shows the number of observations that can be explained by a specific combination of conditions but not by another combination of conditions, and consistency, which indicates the reliability or fitness of the paths (conditions or combination of conditions) that explain the observed results.

## 4. Results

### 4.1. PLS-SEM results

In this study, we assessed the overall impact of the causal path in the hypothetical model.

#### 4.1.1. Evaluation of measurement models

Our model evaluation revealed significant loadings for the items measuring neighborhood environment, bedtime habits, stress, and self-control, supporting our hypothesized model. The model fit was satisfactory. The SRMR is <0.1, indicating a good model fit (Chen et al., 2008). The structural reliability of all variables, including neighborhood environment, bedtime habits, stress, self-control, and sleep quality, was acceptable. Cronbach’s Alpha coefficient for all variables was higher than 0.6, meeting the standard recommended by Cohen (2013). The combination reliability was greater than 0.7, indicating the stable internal consistency of each dimension (Shrestha, 2021). The average variance extracted (AVE) exceeded the accepted threshold of 0.4 (Fornell and Larcker, 1981), demonstrating the effectiveness of the convergence. Concerning discriminant validity, the correlation between each variable was lower than the square root of the extracted average variance (Henseler et al., 2015), thereby confirming the discriminant validity of the findings (Table 3).

**Table 3:** Evaluation of measurement models.

Reliability and convergent validity				Discriminant validity				
AVE	CR	Cronbach’s alpha		S	BH	PSQI	SC	ND
0.455	0.806	0.705	S	0.674				
0.456	0.768	0.613	BH	0.36	0.675			
1	1	1	PSQI	0.294	0.065	1		
0.775	0.874	0.711	SC	0.575	0.322	0.287	0.881	
0.837	0.912	0.807	ND	0.069	−0.228	0.088	0.077	0.915

S, stress; BH, bedtime habits; PSQI, sleep quality; SC, self-control; ND, neighborhood environment.

#### 4.1.2. Evaluation of structural models

The *t*-values of the proposed relationship paths supported hypotheses H1, H2, H4, H5, H7, and H9, while rejecting hypotheses H3, H6, and H8 (Table 4). Specifically, stress and self-control had a positive and significant impact on sleep quality, supporting H1 (*t*-value = 2.555, *p* < 0.05), H5 (*t*-value = 2.518, *p* < 0.05), H10 (*t*-value = 8.926, *p* < 0.05), and H11 (*t*-value = 8.734, *p* < 0.05); bedtime habits, stress, and self-control completely mediated the relationship between neighborhood environment and sleep quality, supporting H2 (*t*-value = 5.737, *p* < 0.05), H4 (*t*-value = 5.332, *p* < 0.05), H7 (*t*-value = 2.771, *p* < 0.05), and H9 (*t*-value = 2.457, *p* < 0.05). However, bedtime habits did not have a significant effect on sleep quality (H3: *t*-value = 0.769, *p* > 0.05), and neither did neighborhood environment on stress (H6: *t*-value = 1.618, *p* > 0.05) or on sleep quality (H8: *t*-value = 0.658, *p* > 0.05). An indirect effect occurs when the influence of one latent variable on another variable is mediated, either wholly or partially, by one or more intervening variables. An indirect effect as significant when all the individual paths involved in that indirect effect were significant (Leth-Steensen and Gallitto, 2015).

**Table 4:** Evaluation of structural models.

Relationships	*t*-value	*p-*value	Conclusion
S - > PSQI(H1)	2.555	0.011	Support
BH - > S(H2)	5.737	0.000	Support
BH - > PSQI(H3)	0.769	0.442	Non-support
BH - > SC(H4)	5.332	0.000	Support
SC - > PSQI(H5)	2.518	0.012	Support
ND - > S(H6)	1.618	0.106	Non-support
ND - > BH(H7)	2.771	0.006	Support
ND - > PSQI(H8)	0.658	0.511	Non-support
ND - > SC(H9)	2.457	0.014	Support
S- > SC(H10)	8.926	0.000	Support
SC- > S(H11)	8.734	0.000	Support

S, stress; PSQI, sleep quality; BH, bedtime habits; SC, self-control; ND, neighborhood environment.

### 4.2. fsQCA results

In this study, fsQCA was used to describe each sample as a combination of antecedent variables and outcomes. The outcome variable in our study was sleep quality, and the antecedent variables were factors that might affect sleep quality, including stress, self-control, bedtime habits, and neighborhood environment.

#### 4.2.1. Necessary condition analysis

The results obtained (Table 5) indicate that there are no necessary conditions for satisfaction since the consistency is less than 0.90 in all cases (Ragin, 2009).

**Table 5:** Necessary condition analysis.

Outcome variable: PSQI		
Conditions tested:		
	Consistency	Coverage
S	0.612111	0.607288
~S	0.723555	0.725323
SC	0.668502	0.744178
~SC	0.649312	0.586447
ND	0.721982	0.672282
~ND	0.607943	0.652596
BH	0.701062	0.675252
~BH	0.630908	0.652248
Outcome variable:~PSQI		
Conditions tested:		
	Consistency	Coverage
S	0.727493	0.725734
~S	0.606336	0.611163
SC	0.544623	0.609613
~SC	0.771451	0.700597
ND	0.678139	0.634933
~ND	0.649981	0.701562
BH	0.665468	0.644497
~BH	0.664685	0.690950

S, stress; SC, self-control; ND, neighborhood environment; BH, bedtime habits; PSQI, sleep quality.

#### 4.2.2. Sufficiency analysis

The model achieved an overall consistency of 0.75 and an overall coverage of 0.50, which met the minimum standards for consistency and coverage (An et al., 2020). The intermediate solutions were recommended as several combinations of antecedent conditions were found to affect sleep quality (Ragin, 2009). The first pathway indicated that sleep quality was affected by bedtime habits, self-control, and stress (consistency = 0.826, coverage = 0.480), while the second pathway indicated that sleep quality was affected by neighborhood environment, self-control, and stress (consistency = 0.832, coverage = 0.494) (Table 6).

**Table 6:** fsQCA results for predicting sleep quality.

	RC	UC	C		RC	UC	C
Causal models of good sleep quality				Causal models of poor sleep quality			
PSQI = f(S, SC, ND, BH)				~PSQI = f(S, SC, ND, BH)			
C1:~S*SC*ND	0.490129	0.0866693	0.835276	C1:S* ~ SC	0.638404	0.154791	0.77131
C2:~S*SC*BH	0.475737	0.0722769	0.829425	C2:~SC* ~ ND* ~ BH	0.437153	0.0408292	0.85945
				C3:S* ~ ND*BH	0.439577	0.0394995	0.815083
Solution coverage: 0.562406				Solution coverage: 0.718733			
Solution consistency: 0.820633				Solution consistency: 0.764031			
							
Causal models of good self-control				Causal models of poor self-control			
SC = f(BH, ND)				~SC = f(BH, ND)			
C1:BH*ND	0.585974	0.585974	0.755758	C1:~BH	0.664796	0.664796	0.760956
Solution coverage: 0.585974				Solution coverage: 0.664796			
Solution consistency: 0.755758				Solution consistency:0.760956			
							
Causal models of high stress				Causal models of low stress			
S = f(BH, ND)				~S = f(BH, ND)			
C1:~BH* ~ ND	0.495162	0.495162	0.846133	C1:BH*ND	0.579391	0.579391	0.829832
Solution coverage: 0.495162				Solution coverage: 0.579391			
Solution consistency: 0.846133				Solution consistency: 0.829832			
							
Causal models of good sleep quality				Causal models of poor sleep quality			
PSQI = f(SC, S)				~PSQI = f(SC, S)			
C1:SC* ~ S	0.582383	0.582383	0.805942	C1:~SC*S	0.638404	0.638404	0.77131
Solution coverage: 0.582383				Solution coverage: 0.638404			
Solution consistency: 0.805942				Solution consistency: 0.77131			

RC, raw coverage; UC, unique coverage; C, consistency; S, stress; SC, self-control; ND, neighborhood environment; BH, bedtime habits; PSQI, sleep quality.

The causal analysis indicated that bedtime habits and neighborhood environment influenced self-control (consistency = 0.756, coverage = 0.586) (Table 6). Additionally, the analysis showed that bedtime habits and neighborhood environment had an impact on stress (consistency = 0.835, coverage = 0.579) (Table 6). The analysis of the causal relationship between self-control, stress, and sleep quality revealed that good self-control and low stress were associated with improved sleep quality (consistency = 0.802, coverage = 0.587) (Table 6).

## 5. Discussion

This study expands on the existing research on factors that impact the sleep quality of college students. We investigated the factors related to stress, self-control, bedtime habits, and neighborhood environment, utilizing complementary methodologies of PLS-SEM and fsQCA models.

**Figure 2:**
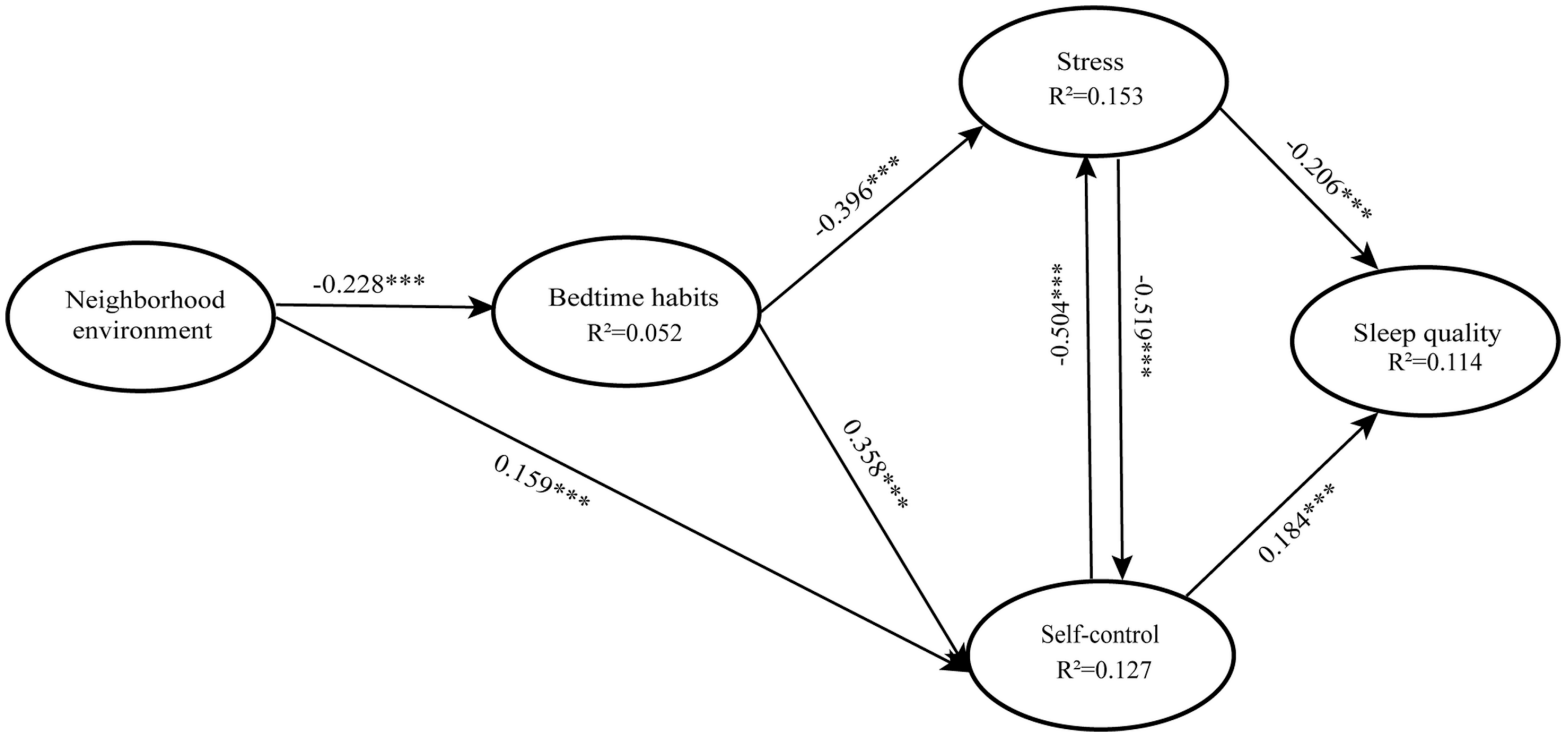
Results for a structural model. *** Statistically significant relationship, *p ≤ 0.05, The diagram presents all significant relationship paths.

Based on the literature, sleep quality is primarily influenced by perceived stress (Yun and Greenwood, 2022) and self-regulatory abilities (Lin and Chung, 2022). The PLS-SEM models demonstrated that the mediating predictive variables were stress and self-control. The fsQCA model revealed that low stress and high self-control were the necessary conditions for good sleep quality. Stress and self-control are considered key factors affecting the sleep quality of college students. Bedtime habits and neighborhood environment were also found to have an impact on college students’ sleep quality. This hypothesis was supported by the fsQCA model and partially supported by the PLS-SEM model. The results showed the effect of the combination of bedtime habits and neighborhood environment on sleep quality, which significantly enriched the theoretical research on sleep quality (Figure 2). There were multiple causal combinations that could affect sleep quality: a combination of higher stress levels and weaker self-control was an important factor leading to sleep disorders (Table 6). Under poor neighborhood environment and high stress, even with better bedtime habits, sleep quality was not improved (Table 6). Good sleep quality not only required better bedtime habits but also demanded lower stress and better self-control (Table 6). These findings confirm that stress, self-control, bedtime habits, and neighborhood environment are fundamental factors affecting the sleep quality of college students. Stress and self-control directly impact the sleep quality, while bedtime habits and neighborhood environment indirectly impact the sleep quality. Additionally, the fsQCA models demonstrate that stress and self-control are crucial conditions for college students’ sleep quality. The literature suggests that dissatisfaction with sleep triggers anxiety (Thakral et al., 2021) and becomes a source of stress and a catalyst for the loss of self-control, resulting in poor sleep quality. There is a significant correlation between self-control and stress perception (Navarro-Mateu et al., 2020), and improving self-control can also help alleviate stress (Navia et al., 2019). Experimental tests and repeated measurements have found that social stress can lead to the failure of self-control (Halfmann and Rieger, 2019), indicating the crucial role of stress and self-control.

This study has practical implications for understanding how different variables interact to influence college students’ sleep quality, utilizing PLS-SEM and fsQCA methodologies. There are few studies that have examined the complex variables that influence college students’ sleep quality using these methods. The PLS-SEM model showed that stress and self-control were the unique predictive variables. The fsQCA model confirmed that stress and self-control were fundamental conditions for good sleep quality. These results suggest that personalized interventions can be implemented to improve sleep quality by focusing on college students’ self-control ability and stress levels. The reduction of stress through psychoeducation and the improvement of self-control through psychological intervention, along with the development of good bedtime habits and a favorable neighborhood environment, are ways to improve the sleep quality of college students. Consuming moderate quantities of low-caffeine green tea (LCGT) has been shown to reduce stress levels and enhance sleep quality (Unno et al., 2017). Soothing music has also demonstrated the ability to reduce stress, leading to improved perceived sleep quality, longer sleep duration, higher sleep efficiency, and other related benefits (Petrovsky et al., 2021). This is consistent with our findings that conditions such as bedtime habits and sleep environment can affect sleep quality through stress, and the sleep quality can be improved by reducing stress. Mindfulness practices can also contribute to enhancing self-control, leading to improved sleep quality (Liu et al., 2018). This is consistent with our findings that sleep quality can be improved by enhancing self-control. This study is a useful first step toward understanding the complex factors influencing college students’ sleep quality.

Despite its significance, this study has several limitations. Firstly, the study was conducted using a convenience sample from a single university, which limits the generalizability of the findings. Future research should aim to use larger and more diverse samples from multiple universities. Secondly, the study examined only a limited number of factors affecting sleep quality, and further research is needed to explore the causal relationships between more influencing factors. Thirdly, Due to the variations in experiences, challenges facing, and perceived stress among different populations, it is necessary to assess and categorize the stress levels of different groups in future research and discuss, respectively, on the sleep quality of different populations. In future studies, it is important to consider the broader implications of sleep health for both individuals and society as a whole. Future studies could expand the target audience and explore other factors that affect sleep quality to better meet the needs of society.

## 6. Conclusion

Our study contributes to the sleep literature by investigating the predictors of sleep quality in college students, including stress, self-control, bedtime habits, and neighborhood environment, which provides a new perspective on the complex factors influencing sleep quality. The combination of PLS-SEM and fsQCA methods generated novel insights, revealing that stress and self-control are direct factors affecting sleep quality, while neighborhood environment and bedtime habits indirectly affect sleep quality through the mediating effects of stress and self-control. Furthermore, we found that bedtime habits and neighborhood environment can be combined with stress and self-control, respectively, to influence sleep quality. Our study also identified multiple causal configurations that impact sleep quality, enhancing our understanding of the effective pathways through which factors influence sleep quality and providing valuable insights for developing strategies to improve sleep quality in college students.

## Data availability statement

The raw data supporting the conclusions of this article will be made available by the authors, without undue reservation.

## Ethics statement

The studies involving human participants were reviewed and approved by Ethics Committee of Zhejiang Shuren University, Hangzhou, China. The patients/participants provided their written informed consent to participate in this study.

## Author contributions

XD, JZ, ZX, and JL: conceived the project with the input of YW and KC. YW and XD: writing. JZ and ZX: literature searching. JL: compilation and distribution of questionnaires. YW, XD, JZ, ZX, and JL: construction of model. All authors contributed to the article and approved the submitted version.

## Funding

This study was funded by several sources, including the Provincial Industry-University Cooperation Collaborative Education Project (NO.318 [2022] of the Zhejiang Development Reform Society), the Scientific and Technological Innovation Activity Plan and New Seedling Talent Plan for College Students in Zhejiang Province in 2023(NO.2023R420026), the First-class Curriculum Project of Zhejiang Province of China (NO.195 [2021] of the Zhejiang Education Office Letter), the First-class Curriculum Project of Zhejiang Province of China (NO.352 [2022] of the Zhejiang Education Office Letter), the First Batch of Ideological and Political Demonstration Courses of Zhejiang Province of China (NO.47 [2021] of the Zhejiang Education Letter), and the High-level Pre-level Program of Zhejiang Shuren University in 2019.

## Conflict of interest

The authors declare that the research was conducted in the absence of any commercial or financial relationships that could be construed as a potential conflict of interest.

## Publisher’s note

All claims expressed in this article are solely those of the authors and do not necessarily represent those of their affiliated organizations, or those of the publisher, the editors and the reviewers. Any product that may be evaluated in this article, or claim that may be made by its manufacturer, is not guaranteed or endorsed by the publisher.
