# Supplementary material for: What complex factors influence sleep quality in college students? PLS-SEM vs. fsQCA
Source: Front Psychol. 2023 Aug 25;14:1185896. doi: 10.3389/fpsyg.2023.1185896 (PMC10485266; doi:10.3389/fpsyg.2023.1185896)
Supplement: Supplementary file 1 [file Data_Sheet_1.docx]

Supplementary Material

What Complex Factors Influence Sleep Quality in College Students? PLS-SEM vs. fsQCA

Ying Wang^1,†^, Xinyi Dai^1,†^, Jie Zhu^2^, Zeling Xu^3^, Jiayao Lou^4^, Keda Chen^*^

*** Correspondence:** Keda Chen: chenkd@zjsru.edu.cn

# Supplementary Data

Pittsburgh Sleep Quality Index (PSQI)

Instructions:

The following questions relate to your usual sleep habits during the past month only. Your answers should indicate the most accurate reply for the majority of days and nights in the past month. Please answer all questions.

1. During the past month, when have you usually gone to bed at night?

USUAL BED TIME

2. During the past month, how long (in minutes) has it usually take you to fall asleep each night?

NUMBER OF MINUTES

3. During the past month, when have you usually gotten up in the morning?

USUAL GETTING UP TIME

4. During the past month, how many hours of actual sleep did you get at night? (This may be different than the number of hours you spend in bed.)

HOURS OF SLEEP PER NIGHT

For each of the remaining questions, check the one best response. Please answer all questions.

5. During the past month, how often have you had trouble sleeping because you...

(a) Cannot get to sleep within 30 minutes

Not during the past month

Less than once a week

Once or twice a week

Three or more times a week

(b) Wake up in the middle of the night or early morning

Not during the past month

Less than once a week

Once or twice a week

Three or more times a week

(c) Have to get up to use the bathroom

Not during the past month

Less than once a week

Once or twice a week

Three or more times a week

(d) Cannot breathe comfortably

Not during the past month

Less than once a week

Once or twice a week

Three or more times a week

(e) Cough or snore loudly

Not during the past month

Less than once a week

Once or twice a week

Three or more times a week

(f) Feel too cold

Not during the past month

Less than once a week

Once or twice a week

Three or more times a week

(g) Feel too hot

Not during the past month

Less than once a week

Once or twice a week

Three or more times a week

(h) Had bad dreams

Not during the past month

Less than once a week

Once or twice a week

Three or more times a week

(i) Have pain

Not during the past month

Less than once a week

Once or twice a week

Three or more times a week

(j) Other reason(s), please describe

How often during the past month have you had trouble sleeping because of this?

Not during the past month

Less than once a week

Once or twice a week

Three or more times a week

6. During the past month, how would you rate your sleep quality overall?

Very good

Fairly good

Fairly bad

Very bad

7. During the past month, how often have you taken medicine (prescribed or “over the counter”) to help you sleep?

Not during the past month

Less than once a week

Once or twice a week

Three or more times a week

8. During the past month, how often have you had trouble staying awake while driving, eating meals, or engaging in social activity?

Not during the past month

Less than once a week

Once or twice a week

Three or more times a week

9. During the past month, how much of a problem has it been for you to keep up enough enthusiasm to get things done?

No problem at all

Only a very slight problem

Somewhat of a problem

A very big problem

10. Do you have a bed partner or roommate?

No bed partner or roommate

Partner/roommate in other room

Partner in same room, but not same bed

Partner in same bed

If you have a roommate or bed partner, ask him/her how often in the past month you have had...

(a) Loud snoring

Not during the past month

Less than once a week

Once or twice a week

Three or more times a week

(b) Long pauses between breaths while asleep

Not during the past month

Less than once a week

Once or twice a week

Three or more times a week

(c) Legs twitching or jerking while you sleep

Not during the past month

Less than once a week

Once or twice a week

Three or more times a week

(d) Episodes of disorientation or confusion during sleep

Not during the past month

Less than once a week

Once or twice a week

Three or more times a week

(e) Other restlessness while you sleep: please describe

Not during the past month

Less than once a week

Once or twice a week

Three or more times a week

Scoring Instructions for the Pittsburgh Sleep Quality Index

The Pittsburgh Sleep Quality Index (PSQI) contains 19 self-rated questions and 5 questions rated by the bed partner or roommate (if one is available). Only self-rated questions are included in the scoring. The 19 self-rated items are combined to form seven “component” scores, each of which has a range of 0-3 points. In all cases, a score of “0” indicates no difficulty, while a score of “3” indicates severe difficulty.

The seven component scores are then added to yield one “global” score, with a range of 0-21 points,“0” indicating no difficulty and “21” indicating severe difficulties in all areas.

Scoring proceeds as follows:

Component 1: Subjective sleep quality

Examine question #6, and assign scores as follows:

Response Component 1 score

“Very good” 0

“Fairly good” 1

“Fairly bad” 2

‘Very bad” 3

Component 1 score:

Component 2: Sleep latency

1. Examine question #2, and assign scores as follows:

Response Score

≤15 minutes 0

16-30 minutes 1

31-60 minutes 2

＞ 60 minutes 3

Question #2 score:

2. Examine question #5a, and assign scores as follows:

Response Score

Not during the past month 0

Less than once a week 1

Once or twice a week 2

Three or more times a week 3

Question #5a score:

3. Add #2 score and #5a score

Sum of #2 and #5a:

4. Assign component 2 score as follows:

Sum of #2 and #5a Component 2 score

0 0

l-2 1

3-4 2

5-6 3

Component 2 score:

Component 3: Sleep duration

Examine question #4, and assign scores as follows:

Response Component 3 score

＞7 hours 0

6-7 hours 1

5-6 hours 2

＜5 hours 3

Component 3 score:

Component 4: Habitual sleep efficiency

(1) Write the number of hours slept (question # 4) here:

(2) Calculate the number of hours spent in bed:

(Getting up time (question # 3)- Bedtime (question # 1))/ Number of hours spent in bed:

(3) Calculate habitual sleep efficiency as follows:

(Number of hours slept/Number of hours spent in bed) X 100 = Habitual sleep efficiency (%)

( / )X100= %

(4) Assign component 4 score as follows:

Habitual sleep efficiency % Component 4 score

＞85% 0

75-64% 1

65-74% 2

＜65% 3

Component 4 score:

Component 5: Sleep disturbances

(1) Examine questions # 5b-5j, and assign scores for each question as follows:

Response Score

Not during the past month 0

Less than once a week 1

Once or twice a week 2

Three or more times a week 3

#5b score

c score

d score

e score

f score

g score

h score

i score

j score

(2) Add the scores for questions # 5b-5j:

Sum of # 5b-5j:

(3) Assign component 5 score as follows:

Sum of # 5b-5j Component 5 score

0 0

1-9 1

10-16 2

19-27 3

Component 5 score:

Component 6: Use of sleeping medication

Examine question # 7 and assign scores as follows:

Response Component 6 score

Not during the past month 0

Less than once a week 1

Once or twice a week 2

Three or more times a week 3

Component 6 score:

Component 7: Daytime dystunction

(1) Examine question # 8, and assign scores as foflows:

Response Score

Never 0

Once or twice 1

Once or twice each week 2

Three or more times each week 3

Question #8 score:

(2) Examine question t 9, and assign scores as follows:

Response Score

No problem at all 0

Only a very slight problem 1

Somewhat of a problem 2

A very big problem 3

Question # 9 score:

(3) Add the scores for question # 8 and # 9:

Sum of #8 and #9:

(4) Assign component 7 score as follows:

Sum of #8 and #9 Component 7 score

0 0

l-2 1

3-4 2

5-6 3

Component 7 score:

Global PSQI Score

Add the seven component scores together:

Global PSGI Score:
